# Weighted single-step genome-wide association study for direct and maternal genetic effects associated with birth and weaning weights in sheep

**DOI:** 10.1038/s41598-024-63974-0

**Published:** 2024-06-07

**Authors:** Hava Khazaei-Koohpar, Mohsen Gholizadeh, Seyed Hasan Hafezian, Seyed Mehdi Esmaeili-Fard

**Affiliations:** 1 Department of Animal Science and Fisheries, Sari Agricultural Sciences and Natural Resources University (SANRU), Sari, Iran; 2 Center for Immunity and Immunotherapy, Seattle Children’s Research Institute, Seattle, USA

**Keywords:** Animal breeding, Genetic association study

## Abstract

Body weight is an important economic trait for sheep meat production, and its genetic improvement is considered one of the main goals in the sheep breeding program. Identifying genomic regions that are associated with growth-related traits accelerates the process of animal breeding through marker-assisted selection, which leads to increased response to selection. In this study, we conducted a weighted single-step genome-wide association study (WssGWAS) to identify potential candidate genes for direct and maternal genetic effects associated with birth weight (BW) and weaning weight (WW) in Baluchi sheep. The data used in this research included 13,408 birth and 13,170 weaning records collected at Abbas-Abad Baluchi Sheep Breeding Station, Mashhad-Iran. Genotypic data of 94 lambs genotyped by Illumina 50K SNP BeadChip for 54,241 markers were used. The proportion of variance explained by genomic windows was calculated by summing the variance of SNPs within 1 megabase (Mb). The top 10 window genomic regions explaining the highest percentages of additive and maternal genetic variances were selected as candidate window genomic regions associated with body weights. Our findings showed that for BW, the top-ranked genomic regions (1 Mb windows) explained 4.30 and 4.92% of the direct additive and maternal genetic variances, respectively. The direct additive genetic variance explained by the genomic window regions varied from 0.31 on chromosome 1 to 0.59 on chromosome 8. The highest (0.84%) and lowest (0.32%) maternal genetic variances were explained by genomic windows on chromosome 10 and 17, respectively. For WW, the top 10 genomic regions explained 6.38 and 5.76% of the direct additive and maternal genetic variances, respectively. The highest and lowest contribution of direct additive genetic variances were 1.37% and 0.42%, respectively, both explained by genomic regions on chromosome 2. For maternal effects on WW, the highest (1.38%) and lowest (0.41%) genetic variances were explained by genomic windows on chromosome 2. Further investigation of these regions identified several possible candidate genes associated with body weight. Gene ontology analysis using the DAVID database identified several functional terms, such as translation repressor activity, nucleic acid binding, dehydroascorbic acid transporter activity, growth factor activity and SH2 domain binding.

Growth-related traits such as body weight are economically important traits in sheep. Body weight measurements of lambs are performed at predefined times, including birth and weaning, so that breeding programs for these traits can be considered. Birth weight is generally associated with growth-related traits and body weights at later ages^1^. Weaning weight is a criterion for animal selection. For example, the total weaning weight of lambs per parity for each ewe is one of the important economic traits that reflects several reproductive traits, including maternal abilities and offspring survival. The phenotypic expression of growth-related traits in lambs, such as birth weight and the early growth rate, specially until weaning age, are determined not only by offspring own genetic potential but also by the maternal environment to provide an appropriate environment in the form of suitable feeding. The environmental component may be divided into permanent and temporary environmental parts. It is strongly recommended that the direct additive, maternal additive, and maternal permanent environmental effects should be included in animal models when evaluating the genetic merit to obtain accurate estimates of variance–covariance components^2^.

With the rapid advancement of genome sequencing technologies and the advent of dense marker BeadChips, it is now possible to determine the genotype of tens of thousands of markers and to conduct association studies between genetic markers and phenotypic records. These types of studies, referred to as genome-wide association studies (GWAS), have become a common approach to precisely identify quantitative trait loci (QTL)^3^.

In several previous GWAS related to growth traits in sheep, associated genetic markers or candidate genes for body weight have been reported^4–9^. However, the genetic basis of sheep breeds or populations, the varied number of samples used for GWAS, and the different densities of genetic marker panels led to different reports for previous studies.

Among the various approaches proposed for GWAS, the weighted single-step GWAS (WssGWAS)^10^ is recently used in populations where a large number of individuals are phenotyped over several generations, but genotyped for a smaller number of individuals who belong usually to the more recent generations. In this method, using phenotypes, genotypes, and a combination of a pedigree-derived relationship matrix and genomic relationship matrix (H matrix), genome-wide estimated breeding values (GEBV) are obtained from a genome-wide single-step best linear unbiased prediction (ssGBLUP,^11^). In the next step, based on the equivalence between marker effect models and breeding value models, GEBVs are converted to marker effects, and SNP effects are consequently estimated. One of the attractive features of WssGWAS is that it is possible to consider unequal variances for SNPs which leads to improved accuracy in estimating SNP effects^10^. In livestock populations, with the small number of phenotypes and genotypes, for which QTLs with large effects play an important role in phenotypic variance of target traits, WssGWAS may perform better than traditional GWAS methods^12^. This method has recently been used to identify genomic regions affecting production and reproductive traits in livestock^13–15^. In the present study, we implemented WssGWAS to identify genomic regions and related candidate genes associated with birth and weaning weights in Baluchi sheep. To understand the genetic basis of birth and weaning weights in sheep, we also performed a post GWAS function analyses including GO and KEGG pathway analyses to identify the biological processes and functional terms significantly enriched with identified candidate genes.

## Material and methods

### Phenotypes, genotypes, and pedigree

The phenotypic data used in this research included 13,408 birth weight and 13,170 weaning weight data of Baluchi lambs that were collected at Abbas-Abad Baluchi Sheep Breeding Station located in Mashhad-Iran. The pedigree file encompassed 22,160 animals including 518 sires and 6,283 dams. Details of management and feeding for Baluchi sheep were previously reported by Gholizadeh et al.^16^. The birth weights of lambs were recorded after birth. Subsequently, the lambs were raised until 90 days of age to determine the waning weight of each lamb. Characteristics of the data structure are summarized in Supplementary Table 1.

A total of 94 lambs were genotyped by Illumina 50K SNP BeadChip for 54,241 markers. Genotype data were provided by the animal genetics group of Sari Agriculture Science and Natural Resource University-Iran^17^. SNPs with unknown positions on the Ovine genome, and SNPs located on sex chromosomes were initially excluded from the data. Further quality control for Autosome SNPs was performed by Plink^18^ using the following criteria: individual call rate ≥ 95%; SNP call rate ≥ 95%; minor allele frequency ≥ 0.05; strong Hardy–Weinberg equilibrium violation p-value ≥ 10^−6^. After quality control, 94 lambs, and 41,102 SNPs remained for further analysis. For WssGWAS analysis, missing genotypes were imputed using Beagle software version 4.1^19^.

### Statistical model

First, weaning weights were adjusted to 90 days of age as follows,

$$\left( {\frac{{{\text{unadjusted WW }} - {\text{BW}}}}{{{\text{lamb age }}\left( {{\text{day}}} \right){\text{ at weaning}}}}} \right) \times 90$$

where BW and WW are the birth weight and weaning weight of lambs, respectively. The univariate animal model including maternal effects used for ssGBLUP was as follows:

$${\mathbf{y}} = {\mathbf{X}}{\text{b }} + {\mathbf{Za}} + {\mathbf{Wp}} + {\mathbf{Sm}} + {\mathbf{e}}$$

where **y** is the vector of observations; b is the vector of significant fixed effects (sex, birth type, herd, birth year, and age of mother at lambing); $\mathbf{a}\boldsymbol{ }\sim N(0,{\varvec{H}}{\sigma }_{\text{a}}^{2})$ is the vector of direct additive genetic effects; $\mathbf{p}\boldsymbol{ }\sim N(0,{\varvec{I}}{\sigma }_{\text{p}}^{2})$ is the vector of maternal permanent environmental effects; $\mathbf{m}\boldsymbol{ }\sim N(0,{\varvec{H}}{\sigma }_{\text{m}}^{2})$ is the vector of maternal genetic effects; $\mathbf{e}\boldsymbol{ }\sim N(0,{\varvec{I}}{\sigma }_{\text{e}}^{2})$ is the vector of random residuals; where ${\sigma }_{\text{a}}^{2}$, ${\sigma }_{\text{p}}^{2}$, ${\sigma }_{\text{m}}^{2}$, and ${\sigma }_{\text{e}}^{2}$ are the additive genetic, maternal permanent environmental, maternal genetic and residual variances, respectively. **X**, **Z**, **W** and **S** are the incidence matrices of **b**, **a**,** p**, and **m**, respectively.

**H** is the hybrid matrix that combines pedigree and genomic information^11,20^, and **I** is an identity matrix. The inverse of the H matrix was calculated as follows,

$${\varvec{H}}^{ - 1} = {\varvec{A}}^{ - 1} + \left| {\begin{array}{*{20}c} 0 & 0 \\ 0 & {{\varvec{G}}_{\omega }^{ - 1} - {\varvec{A}}_{22}^{ - 1} } \\ \end{array} } \right|$$

where A is the numerator relationship matrix based on pedigree data for all individuals; A_22_ is the numerator relationship matrix corresponding to the genotyped individuals; ***G***_*ω*_ is the weighted genomic relationship matrix which was obtained using ***G***_*ω*_ = *αG* + *βA*_22_ where α and β are weighting factors, selected to be 0.95 and 0.05, respectively. These weights were used to make G positive-definite, improve convergence^21,22^ , scale the genomic information to be compatible with the pedigree information and to control bias^23,24^; G is the genomic relationship matrix^21^, obtained as follows:

$$G = \frac{ZDZ^{\prime}}{{\mathop \sum \nolimits_{i = 1}^{m} 2p_{i} \left( {1 - p_{i} } \right)}}$$

where *Z* is a matrix of gene content adjusted for allele frequencies (with elements of 0-2p, 1-2p, and 2-2p representing genotypes *AA*, *Aa*, and *aa*, respectively; *p* is the minor allele frequency (MAF)), *D* represents a diagonal matrix carried the weights of SNP, *p*_i_ is the MAF of the *i*^th^ SNP and *m* implies the number of SNPs.

Variance components and heritability of the studied traits were estimated using average information restricted maximum likelihood (AI-REML) method^25^ via pedigree data. Marker effects and further weights required for constructing G matrix were calculated in an iterative way proposed by Wang et al.^10,26^. An iteration method with the steps described below was used for the association study.

1. First iteration (t = 1),
  $$\left( {{\text{t }} = { 1}} \right):{\mathbf{D}} = {\mathbf{I}};{\mathbf{G}}\left( {\text{t}} \right) \, = {\mathbf{ZD}}({\mathbf{t}}){\mathbf{Z}}\prime ,\;where\lambda = \frac{1}{{\mathop \sum \nolimits_{i = 1}^{M} 2p_{i} \left( {1 - p_{i} } \right)}} \left( {{\text{VanRaden}},{ 2}00{8}} \right)$$
2. GEBV calculation,
  In this step, GEBVs were calculated for the entire population using the ssGBLUP approach, considering:
  $$\mathbf{H}^{{ - 1}} = \mathbf{A}^{{ - 1}} + \left| {\begin{array}{*{20}c} {\mathbf{0}} & {\mathbf{0}} \\ {\mathbf{0}} & {\left( {0.95\mathbf{G}_{{\left( t \right)}} + 0.05\mathbf{A}_{{22}} } \right)^{{ - 1}} - \mathbf{A}_{{22}}^{{ - 1}} } \\ \end{array} } \right|$$
3. Calculation of marker effects,
  Marker effects ($\widehat{{\varvec{u}}}$) were obtained via GEBV conversion as,$\varvec{\hat{u}}(t) = \lambda \varvec{D}_{{(t)}} \varvec{Z}\prime \varvec{G}_{{(t)}}^{{ - 1}} \widehat{\varvec{a}}_{g}$ where ${\widehat{a}}_{g}$ is the GEBV of individuals that were genotyped.
4. Calculation of SNP weights,
  SNP weights for the next iteration were obtained using the $\text{Non}$ linearA approach as follows, ${d}_{i}= {CT}^{\frac{ \left|{\widehat{a}}_{i}\right|}{sd(\widehat{a})}-2}$^21^. where $CT$ is a constant, (1.125,^21^), that determines the departure from normality; $\left|{\widehat{a}}_{i}\right|$ is the estimated effect for marker *j*, and $sd(\widehat{a})$ is the standard deviation of the estimated effects of SNP. The NonlinearA approach avoids extreme values for weights and provides good convergence properties^27^. This is achieved as the maximum variation in weights is limited by the minimum between 5 and the exponent of CT^28^.
5. The normalization of SNP weights to keep the total genetic variance constant as follows:
  $$D_{{\left( {t + 1} \right)}} = \frac{{tr\left( {D_{t} } \right)}}{{tr\left( {D_{t} + 1} \right)}} D_{{\left( {t + 1} \right)}}$$
6. Weighted G calculation (${G}_{(t+1}$) as follows,
  $${G}_{(t+1)}= \lambda Z{D}_{(t+1)}Z{\prime}$$
7. t = t + 1 and loop to step 2.
  The SNP effects were estimated by 3 iterations. The percentage of genetic variance explained by the i^th^ SNP window was calculated as follows:
  $$\frac{{var\left( {a_{i} } \right)}}{{\sigma_{a}^{2} }} \times 100\% = \frac{{var\left( {\mathop \sum \nolimits_{j = 1}^{x} Z_{j} \hat{u}_{j} } \right)}}{{\sigma_{a}^{2} }} \times 100\%$$
  where ai is the genetic value of the ith SNP window that consists of a region of consecutive SNPs; ${\sigma }_{a}^{2}$ is the total additive genetic variance; ${Z}_{j}$ is the vector of the gene content of the *j*th SNP for all individuals; and ${\widehat{u}}_{j}$ is the effect of the *j*th SNP within the *i*th window.

**Figure 1:**
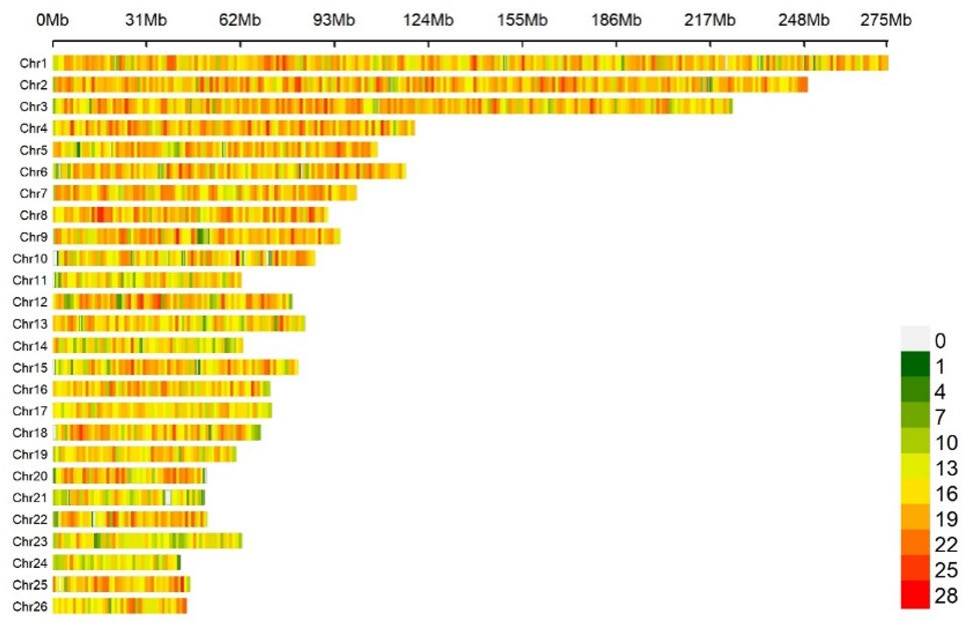
The SNP-density plot representing the number of quality-passed SNPs within each 1Mbp window size.

The iteration steps above were conducted via the BLUPF90 software family^23^for genomic analyses^29^. Initially, variance components were estimated using AIREMLF90 under a univariate animal model considering maternal effects. Estimated variance components then used in BLUPF90 for GEBVs prediction. PostGSf90 software was then used for SNP effects calculation. The proportion of variance explained by non-overlapping windows was estimated by summing the variance of SNPs within 1 megabase (Mb)^30,31^. SNP-density plot created by the CMplot package^32^ representing the number of quality-passed SNPs within each 1Mbp window size is given in Fig. 1. The Top 10 window regions explaining the highest percentages of additive and maternal genetic variance were selected as candidate QTL regions associated with body weights as used in previous studies in animal species^31,33^. Manhattan plots showing the distribution of marker effects over the genomic position were created using the CMplot package^32^ in R^34,35^.

### Candidate gene identification and gene set enrichment analysis

Candidate genes associated with significant windows were retrieved from the NCBI Genome Data Viewer (https://www.ncbi.nlm.nih.gov/genome/gdv/) using OAR4 assembly. To identify Molecular functions and pathways, gene set enrichment analysis was conducted following wssGWAS analysis using DAVID (david.ncifcrf.gov/). GO terms and KEGG signaling pathways with a Fisher Exact *P*-value ≤ 0.01 were reported as significant.

## Results

In this study, we identified window genomic regions associated with body weight at birth and weaning in the Baluchi sheep breed using WssGWAS.

### Birth weight

The top 10 significant SNP windows that contributed to direct and maternal genetic effects for birth weight along with the identified potential candidate genes are listed in Table 1. The top 10 SNP windows explained a total of 4.30% of direct additive genetic variance. The additive genetic variance explained by the genomic window regions varied from 0.59 on chromosome 8 (37,942,516–38,934,945) to 0.31 on chromosome 1 (214,063,075–215,052,580) (Supplementary Tables 2–4). Further investigation of these regions on the NCBI Genome Data Viewer identified many genes in these areas.

**Table 1:** The top 10 significant genomic windows and candidate genes contributing to the direct additive and maternal genetic effects for birth weight in the Baluchi sheep.

chr	Start	End	Variance explained%	Genes
Direct additive				
8	37,904,980	38,889,352	0.58771	FBXL4
10	41,788,680	42,735,479	0.56467	–
2	70,799,737	71,770,895	0.51754	GLIS3, RFX3
4	92,470,191	93,383,584	0.48573	AHCYL2 ,ATP6V1F,CALU,CCDC136, FAM71F1, FAM71F2, FLNC ,HILPDA, IMPDH1, IRF5, KCP , LEP,OPN1SW , PRRT4 ,RBM28, SMO, STRIP2, TNPO3, TSPAN33
7	17,151,460	18,138,449	0.44301	LARP6, LRRC49, THAP10, THSD4, UACA
21	20,765,892	21,740,546	0.4058	CCDC179, FANCF, GAS2, SLC17A6, SVIP
24	33,205,900	34,202,715	0.35048	ABHD11,BCL7B, BAZ1B, CCL24, CCL26, CLDN3, CLDN4, DNAJC30, ELN, FKBP6, FZD9, HIP1, MDH2, MLXIPL, NSUN5,POM121C , POR, RHBDD2, SRRM3, STX1A, STYXL1,TBL2,TMEM120A, TRIM50, TRNAE-CUC, VPS37D, WBSCR22, WBSCR27, WBSCR28
25	33,597,554	34,580,662	0.32203	ZMIZ1
13	54,068,828	55,054,571	0.31578	ADRM1, CABLES2 , CDH4, HRH3, LAMA5, LSM14B, MTG2, OSBPL2, PSMA7, RBBP8NL, RPS21, SS18L1, TAF4, TRNAE-UUC
1	213,913,661	214,896,980	0.31136	CLDN11, EIF5A2, RPL22L1, SLC2A2, SLC7A14, TNIK
Maternal				
10	41,788,680	42,735,479	0.83776	–
7	17,151,460	18,138,449	0.69135	LARP6, LRRC49, THAP10, THSD4, UACA
8	37,904,980	38,889,352	0.53859	FBXL4
2	70,799,737	71,770,895	0.49427	GLIS3, RFX3
4	92,526,925	93,489,024	0.39192	AHCYL2, ATP6V1F, CALU, CCDC136, FAM71F1, FAM71F2, FLNC, HILPDA, IMPDH1, IRF5, KCP, NRF1, OPN1SW, PRRT4, RBM28, SMO, STRIP2, TNPO3, TSPAN33
1	209,122,340	210,093,244	0.35262	NAALADL2
25	33,597,554	34,580,662	0.33707	ZMIZ1
5	47,771,920	48,762,985	0.33301	CXXC5,CYSTM1, DNAJC18, ECSCR, IGIP, MATR3, MZB1 ,NRG2, PAIP2, PFDN1, PROB1,PSD2, PURA, SIL1, SLC23A1, SPATA24,TMEM173, UBE2D2
21	20,696,861	21,669,394	0.32567	CCDC179, FANCF, GAS2, SVIP
17	33,635,356	34,621,154	0.32306	BBS12, FGF2, NUDT6, SPATA5, SPRY1

**Figure 2:**
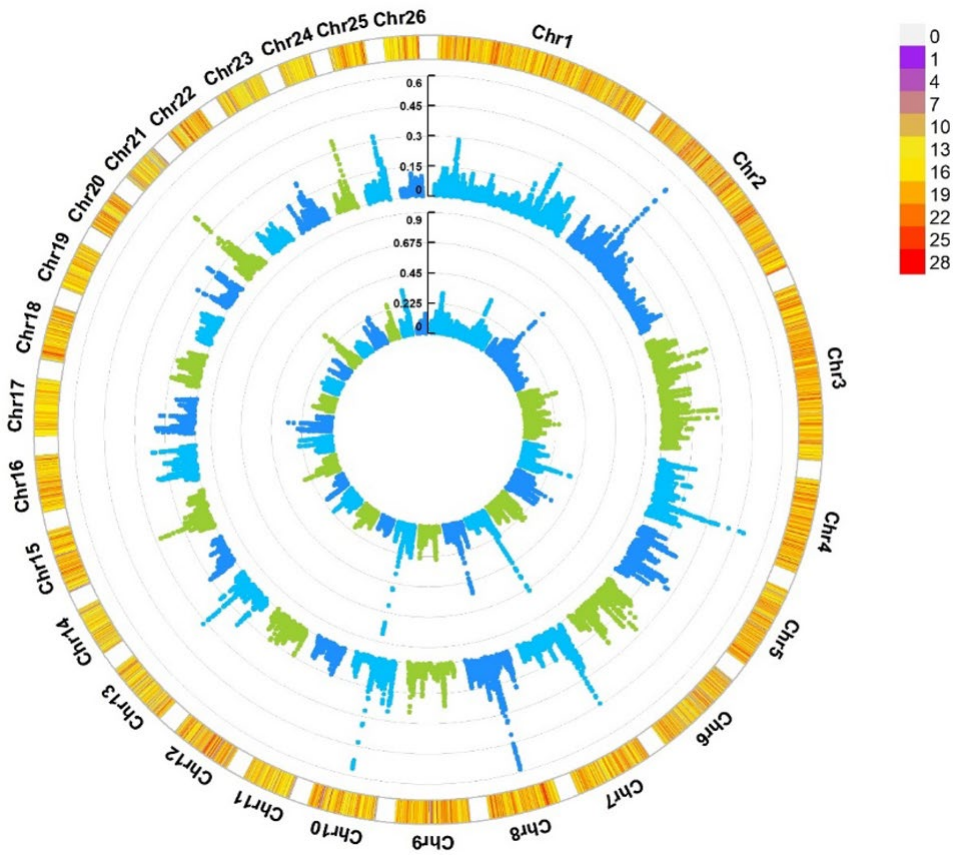
The circular Manhattan plot from the WssGWAS for the direct additive and maternal genetic effects on birth weight explained by SNPs. The 2 circles from inside to outside occupy the maternal and direct additive genetic variance explained by SNPs (%), respectively. The outermost circle shows the SNP density in the 1 Mb window for each chromosome.

For maternal effects on birth weight, a total of 4.62% of maternal genetic variance was explained by the top 10 SNP windows. The highest maternal genetic variance (0.84%) was explained by a genomic window on chromosome 10 (41,802,553- 42,753,634), and the lowest maternal genetic variance (0.32%) was explained by a genomic window on chromosome 17 (33,670,501–34,661,256). In these genomic window regions, several genes for maternal effects were identified (Table 1). Circle Manhattan plot from WssGWAS for direct and maternal genetic effects on birth weight is presented in Fig. 2. Results of gene ontology and KEGG signaling pathway analysis of candidate genes are presented in Table 2

**Table 2:** Significant GO terms and KEGG signaling pathways of candidate genes related to birth weight in Baluchi sheep.

Category	Term	Fisher exact
GOTERM_MF_DIRECT	Translation repressor activity, nucleic acid binding	1.7E−4
	Dehydroascorbic acid transporter activity	2.6E−4
GOTERM_BP_DIRECT	Positive regulation of metallopeptidase activity	1.2E−4
	Lymphocyte proliferation	1.2E−4
	Lung development	1.8E−3
	Mammary gland epithelial cell differentiation	4.2E−4
	Positive regulation of protein phosphorylation	4.0E−3
	Organ induction	7.2E−4
	Negative regulation of wound healing	9.0E−4
	Protein localization to plasma membrane	7.0E−3
	Positive regulation of wound healing	2.1E−3
	Regulation of cell morphogenesis	2.3E−3
	Apoptotic signaling pathway	3.7E−3
	Neural crest cell migration	4.4E−3
GOTERM_CC_DIRECT	Postsynapse	1.7E−3
	Apicolateral plasma membrane	1.6E−3
	Bicellular tight junction	7.8E−3
KEGG_PATHWAY	Huntington disease	7.7E−3
	Cell adhesion molecules	8.1E−3
	Synaptic vesicle cycle	7.2E−3

### Weaning weight

Genomic windows identified for direct genetic and maternal effects that were significantly associated with weaning weight are listed in Table 3. For direct additive genetic effects, the top 10 genomic windows explained a total of 6.4% of the genetic variance. The contribution of explained genetic variance by genomic windows varied from 1.37% on chromosome 2 (235,006,576–235,981,086) to 0.42%, again on chromosome 2 (203,829,884–204,804,430) (Supplementary Tables 5–7). Chromosome 2 with 4 genomic window regions showed the most contribution of additive genetic variance. Several genes were identified for these genomic windows. For instance, the most significant window included LAPTM5, MATN1, SERINC2, FABP3, ZCCHC17, TRNA-CUG, SNRNP40, NKAIN1, PUM1 and SDC3 genes (Table3).

**Table 3:** The top 10 significant genomic windows and candidate genes contributing to the direct additive and maternal genetic effects for weaning weight in the Baluchi sheep.

chr	Start	End	Variance explained%	Genes
Direct additive				
2	235,002,832	235,978,375	1.37	FABP3, LAPTM5, MATN1, NKAIN1, PUM1, SDC3, SERINC2, SNRNP40, TRNA-CUG, ZCCHC17
2	233,630,486	234,608,565	0.92	AK2, AZIN2, BSDC1, CCDC28B, DCDC2B, EIF3I, FAM167B, FAM229A, FNDC5, HDAC1, HPCA, IQCC, KHDRBS1, KIAA1522, KPNA6, LCK, MARCKSL1, RBBP4, RNF19B, S100PBP, SYNC, TMEM39B, TMEM54, TMEM234, TRIM62, TSSK3, TXLNA, YARS, ZBTB8A, ZBTB8B, ZBTB8OS
2	208,923,907	209,916,853	0.60	C2H2orf80, IDH1, PIKFYVE, PLEKHM3, PTH2R
6	12,803,223	13,777,204	0.57	ALPK1, ANK2, AP1AR, C6H4orf32, LARP7, NEUROG2, TIFA, TRNAW-CCA, ZGRF1
1	209,255,075	210,198,899	0.55	NAALADL2
6	33,972,667	34,949,058	0.54	CCSER1,MMRN1, SNCA
3	100,071,013	101,069,435	0.53	AFF3, CHST10, CNOT11, LONRF2, NMS, NPAS2, PDCL3, TBC1D8, RPL31, TRNAC-GCA
12	19,584,227	20,489,556	0.49	RRP15, TGFB2, TRNAC-GCA
24	29,088,916	30,073,791	0.41	WBSCR17
2	203,829,884	204,812,866	0.42	ABI2, CARF, CD28, CTLA4 , FAM117B, ICA1L, NBEAL1 , RAPH1, WDR12
Maternal				
2	235,002,832	235,978,375	1.37701	FABP3, LAPTM5, MATN1, NKAIN1 , PUM1, SDC3, SERINC2, SNRNP40, TRNAQ-CUG, ZCCHC17
2	233,630,486	234,608,565	0.73396	AK2, AZIN2, BSDC1, CCDC28B, DCDC2B, EIF3I, FAM167B, FAM229A, FNDC5, HDAC1, HPCA, IQCC , KHDRBS1, KIAA1522, KPNA6, LCK, MARCKSL, RBBP4, RNF19B, S100PBP, SYNC, TMEM39B, TMEM54, TMEM234, TRIM62, TSSK3, TXLNA, YARS, ZBTB8A, ZBTB8B, ZBTB8OS
10	57,153,830	58,096,880	0.52822	–
1	130,694,009	131,686,819	0.51428	TRNAS-GGA
15	70,344,861	71,299,714	0.4663	–
5	19,721,833	20,686,613	0.44468	ACSL6, CDC42SE2, CSF2, FNIP1, IL3, MEIKIN, RAPGEF6, TRNAC-GCA
7	17,048,100	18,045,914	0.44049	LARP6, LRRC49, THAP10, THSD4, UACA
2	208,923,907	209,916,853	0.42261	C2H2orf80, IDH1,PIKFYVE , PLEKHM3, PTH2R
6	12,803,223	13,777,204	0.41896	ANK2, ALPK1, AP1AR, C6H4orf32, LARP7, NEUROG2, TIFA, TRNAW-CCA , ZGRF1
2	76,878,146	77,868,214	0.41413	PTPRD, TRNAC-GCA

**Figure 3:**
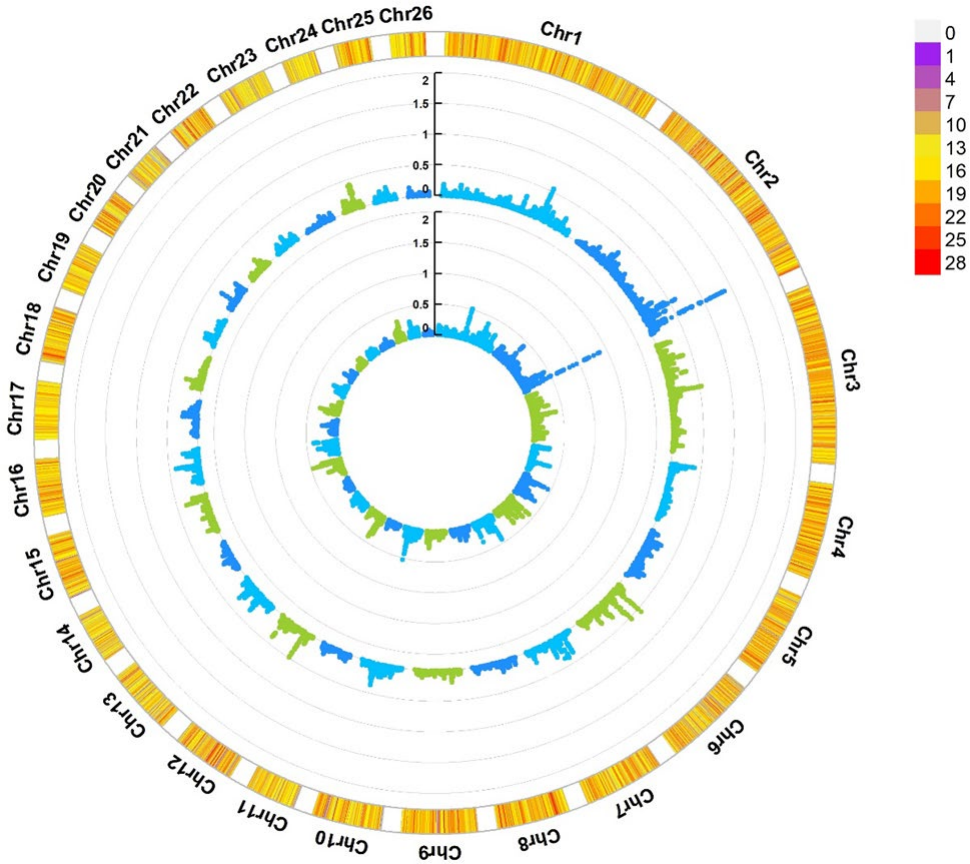
The circular Manhattan plot from the WssGWAS for the direct additive and maternal genetic effects on weaning weight explained by SNPs. The 2 circles from inside to outside occupy the maternal and direct additive genetic variance, respectively. The outermost circle shows the SNP density in the 1 Mb window for each chromosome.

For maternal effects on WW, the highest and lowest maternal genetic variances were explained by genomic windows on chromosome 2. The top 10 window genomic regions explained 5.76% of the maternal genetic variation. The identified genes for the top genomic windows are listed in Table 3. Circle Manhattan plot from WssGWAS for direct and maternal genetic effects on WW is presented in Fig. 3. Significant GO terms and KEGG signaling pathways of candidate genes related to weaning weight are presented in Table 4.

**Table 4:** Significant GO terms and KEGG signaling pathways of candidate genes related to weaning weight in Baluchi sheep.

Category	Term	Fisher exact
GOTERM_MF_DIRECT	Growth factor activity	7.1E−3
	SH2 domain binding	4.0E−3
GOTERM_BP_DIRECT	Cytoplasmic pattern recognition receptor signaling pathway	1.7E−5
	Regulation of T cell proliferation	3.4E−4
	Immune response	7.5E−3
	Negative regulation of viral transcription	1.3E−3
	Positive regulation of I-kappaB kinase/NF-kappaB signaling	7.1E−3
	Apoptotic signaling pathway	3.0E−3
GOTERM_CC_DIRECT	Cytosol	1.2E−3
	Transport vesicle	1.9E−4
	Protein complex involved in cell adhesion	1.4E−4
	Membrane	9.1E−3
	Immunological synapse	4.9E−3
KEGG_PATHWAY	Rheumatoid arthritis	3.1E−4
	T cell receptor signaling pathway	8.4E−4
	Cell adhesion molecules	2.2E−3
	TGF-beta signaling pathway	7.3E−3

## Discussion

In the present study, we identified the top 10 genomic windows that accounted for the highest explained genomic variance as candidate genomic regions for each trait. Studies have shown that the use of sliding windows for simultaneous analysis of multiple SNPs in ssGWAS is able to reduce the noise due to the estimation process^36^. The ssGWAS method combines all genotype, phenotype and pedigree data in single step^10^. Compared with single-marker regression GWAS, this method can use all markers simultaneously, resulting in greater power and precise estimate values^36^. Moreover, ssGWAS utilizes a large amount of phenotypic data of ungenotyped individuals which results in increasing the sample size to a certain extent, improving the accuracy of SNP effect estimation, and increasing the efficiency of SNP identification^37^. However, the ssGWAS model, considers equal variance for all marker effects and therefore may be constrained when traits are influenced by large QTL^10^. To overcome this challenge, the WssGWAS approach was proposed, in which SNP effects are weighted according to their importance in genetic variance of the trait, which improves the precision of QTL detection^10^.

Various studies have shown that growth traits such as body weight at birth and weaning in sheep are affected by direct genetic and maternal effects^16,38^. It has been proven that considering maternal effects in genetic evaluation models leads to a more accurate estimation of (co)variance, genetic parameters and, genetic evaluation of these traits^39^. By using the ssGWAS method, phenotypic and pedigree information of genotyped and non-genotyped animals together with the hybrid relationship matrix leads to a more reliable QTL detection and reducing the probability of spurious signals^40^. In this study, we performed WssGWAS to identify genomic regions and associated candidate genes responsible for genetic variation in BW and WW in Baluchi sheep. The identified genomic regions were associated with 108 and 97 possible candidate genes for BW and WW, respectively among which some of them were previously reported as candidate genes involved in body weight. Promising candidate genes found included AHCYL2, HILPDA, KCP, LEP, FZD9, MDH2, CLDN4, OSBPL2EIF5A2, RPL22L1, RPL22L1, TNIK, CYSTM1, DNAJC18, ECSCR and BBS12 for BW; and FABP3, MATN1, SDC3, FNDC5, PIKfyve, CCSER1, MMRN1, PDCL3, RRP15, TGFB2, RAPH1, ACSL6 and CSF2 for WW.

*AHCYL2* gene has been reported to be associated with the growth, carcass traits, and the meat quality of sheep^41^. *AHCYL2* gene has been reported as the main gene affecting the backfat thickness of beef cattle^42^. It has been documented that body weight in sheep could be controlled through up-regulation of *AHCYL2* gene expression by regulating fat content and muscle development^43^. *HILPDA* is a small, lipid-droplet-associated protein expressed in several tissues that elevate lipid storage in hepatocytes, adipocytes, and macrophages^44^ through directly binding and inhibiting adipose triglyceride lipase^45^. The results of studies on muscle proteomic profiles show that body weight in sheep can be influenced by fat deposition^43^. *KCP* is a secreted protein that regulates the expression and function of BMPs^46^ which are multi-functional growth factors that belong to the transforming growth factor β (TGFβ) superfamily. The roles of BMPs in embryonic development have been extensively reported^47^. Leptin is principally produced in adipose tissue and is engaged in the regulation of body homeostasis, energy intake, storage and expenditure, fertility, and immune functions^48^. Haplotypes of *LEP* genes have been confirmed to be associated with body traits in sheep^49^. Leptin, with respect to its role in the regulation of energy metabolism, has been involved in the harmonious adjustment of maternal adaptations during pregnancy and lactation^50^. It has been reported that human breast milk leptin during the first stages of lactation may regulate processes that affect infant weight gain from the first to the sixth month^51^. *FZD9* has been reported to be associated with lipid metabolism during lactation, quantity of milk produced^52^, pregnancy and, fertility^53^ which can support the role of this gene in the weight of lambs at birth. It has been reported that the *MDH2* gene is less expressed in leaner pig breeds, and also, in general, this gene is expressed at higher levels in females than in males^54^, which could be concluded that this gene is related to body weight. It has been reported that the growth, health and, flexibility of low birth-weight pigs are compromised due to imperfect intestinal development. *CLDN4* is a gene involved in the function of the intestinal barrier, which has a lower expression in pigs with low birth weight, which, along with the delay in the development of intestinal villi and crypts, could be a factor for the growth potential in female piglets with low birth weight^55^. *OSBPL2* is one of the lipid transfer proteins that regulate intracellular cholesterol homeostasis and lipid droplet lipolysis in a way that *OSBPL2* deficiency enlarges intracellular lipid droplets which may lead to obesity^56^. It has been reported that overexpression of *EIF5A2* results in decreased growth rate and body weight in adult transgenic mice^57^. Lower expression of the *RPL22L1* gene, involved in protein synthesis, has been confirmed to be associated with compromised bone formation and growth in the lambs with the most efficient body weight gain^58^. RPL22L1 is also reported as a candidate gene for birth weight in Holstein Friesian^59,60^. It has been demonstrated that TNIK has a critical role in the regulation of energy balance, glucose and fatty acid metabolism, lipid deposition, and insulin sensitivity, and *TNIK* knockout mice show a leaner phenotype. Studies demonstrated that CYSTM1 is significantly associated with the gestation length^61,62^. It has been confirmed that the gestation length is genetically in positive correlation with birth weight^63^. DNAJC18 and ECSCR contributed to maternal genetic effects for birth weight are associated with heat stress in cattle^64,65^. Heat stress during late gestation has been reported to reduce calf birth weight, which last significantly until 7 days of age (Trifkovic 2018). The *BBS12* gene has been reported as a candidate gene for pure meat weight, foreshank weight, and silverside weight in beef cattle^66^.

*FABP3* genes contributed to both direct additive and maternal genetic effects for weaning weight. *FABP3* is involved in fatty acid transport from the cell membrane to the intracellular sites of fatty acid utilization and is mainly expressed in cardiac and skeletal muscle^67^. Clvo et al.^68^ reported a linkage disequilibrium between the *FABP3* gene and a quantitative trait locus for milk fat contents in Manchega breed sheep. It has been reported that the composition of fatty acids in maternal milk fat has a positive effect on the live weight of lambs and the optimization of the growth potential of lambs until weaning^69^. Fatty acids that are produced in the liver and muscles have special functions in the body, and form the primary sources of energy stored in triglycerides, and participate in the formation of complex lipids, hormones and signaling compounds^70^. Cahyadi^71^ reported that *FABP3* was significantly associated with body weights at birth, at 12 to 20 weeks, and slaughter in Korean Native Chickens. Kuzminska^72^ found significant associations between the FABP3 genotypes and the body weight at 210 days of age in Simmental cows. It was reported that there was a significant association between *FABP3* gene variants and body weight and hock weight in swine^73^. The genetic polymorphisms of *FABP3* were associated with early and late-stage stage body weights in the Landrace × Jeju black pig population^74^. It has been reported that the *MATN1* gene plays an important role in muscle growth at different stages of development. The lower expression of this gene is associated with a decrease in muscle growth in low-birth-weight fetuses^75^. Feeding behavior and body weight are regulated by the *SDC3* gene^76^. Srinivasa et al.^77^reported that expression of *FNDC5* in skeletal muscle was associated with mRNA expression of *IGF*-*I* in obese individuals with reduced growth hormone showing a possible function for *FNDC5* in skeletal muscle under a low growth hormone state. It was reported that the PIKfyve pathway is essential in mammary epithelial differentiation during pregnancy^78^ which supports its role in the maternal ability to grow lambs and their weaning weight. It has been reported that CCSER1 is associated with feed efficiency in beef cattle^79^ , growth and feed intake in sheep^80^, knuckle, biceps, and shank of beef carcass traits^81^. MMRN1 has been confirmed to be associated with feed efficiency, growth, and carcass traits in beef cattle^82^. It has been proved that NMS is involved in suckling-induced oxytocin release which is essential for milk ejection in mammals^83^. Lack of oxytocin or its receptor in mice results in deficient milk ejection response to the suckling stimulus^84^. It has been documented that *PDCL3* variants are significantly associated with weight at 12 months of age, carcass weight, and loin eye area in an indigenous Korean cattle breed^85^. *PDCL3* encodes a chaperone protein which through interacting with vascular endothelial growth factor receptor 2 is involved in angiogenesis^86^. *RRP15* has been reported as a candidate gene for milk yield in dairy sheep which is expressed in either the milk transcriptome or the mammary gland^87^. *TGFB2* gene is one of the members of the TGFB superfamily, which plays important roles in embryogenesis, cell differentiation, muscle development, growth, and reproductive regulation^88,89^. The *RAPH1 g*ene has been confirmed to be associated with cell migration and nutrient absorption by rumen epithelia cells^90^. *ACSL6* has been previously reported as a candidate gene associated with dry matter intake and mid-test body weight in the Angus population^91^ . In mammals, ACSL gene is essential for fatty acid degradation, remodeling of the phospholipid, and the long-chain acyl-CoA esters production that regulate different kinds of physiological, metabolism, and cell signaling processes^92^. *CSF2* has been reported as one of the regulatory molecules that mediate maternal effects on embryonic development during the preimplantation period and enhance embryo competence for post-transfer survival^93^.

## Conclusions

In the present study, we conducted a WssGWAS and identified many genomic regions and known Ovine candidate genes for birth weight and weaning weight in sheep which can provide new insights into the genetic basis of growth traits in sheep. These findings can be further investigated to search for causative mutations underlying body weights in lambs and for marker-assisted selection to improve the meat production in sheep. The genetic architecture of growth traits is very complex and these candidate genes may be under breed-specific effects. Therefore, it is recommended to study the genes identified in this study as candidate genes in different breeds of sheep under different environmental conditions. This leads to a better understanding of the complex pathways underlying growth traits in sheep. One limitation of our study is the sample size of genotyped individuals which is not large. Therefore, further high-resolution researches utilizing larger sample sizes may help validate the genomic regions and candidate genes associated with body weight in sheep identified in the present study.

### Supplementary Information

Supplementary Information.

## Data Availability

All data including genotypic data and phenotypes used in the current study that support the findings of this study are available from the corresponding author upon reasonable request. The phenotypes used in the current study were under legal restrictions and commercial ownership set by the Iran National Animal Breeding Center and Promotion of Animal Products and can be made available for academic purposes after signing a Material Transfer Agreement.
